# Workflow assessing the effect of gait alterations on stresses in the medial tibial cartilage - combined musculoskeletal modelling and finite element analysis

**DOI:** 10.1038/s41598-017-17228-x

**Published:** 2017-12-12

**Authors:** K. S. Halonen, C. M. Dzialo, M. Mannisi, M. S. Venäläinen, M. de Zee, M. S. Andersen

**Affiliations:** 1 Department of Health Science and Technology, Aalborg University, Fredrik Bajers Vej 7D, DK-9220 Aalborg, Denmark; 2 Department of Mechanical and Manufacturing Engineering, Aalborg University, Fibigerstræde 16, DK-9220 Aalborg, Denmark; 3 School of Health and Life Science, Glasgow Caledonian University, Cowcaddens Rd, G4 0BA Glasgow, United Kingdom; 4 Department of Applied Physics, University of Eastern Finland, POB 1627, FI-70211 Kuopio, Finland

## Abstract

Knee osteoarthritis (KOA) is most common in the medial tibial compartment. We present a novel method to study the effect of gait modifications and lateral wedge insoles (LWIs) on the stresses in the medial tibial cartilage by combining musculoskeletal (MS) modelling with finite element (FE) analysis. Subject’s gait was recorded in a gait laboratory, walking normally, with 5° and 10° LWIs, toes inward (‘Toe in’), and toes outward (‘Toe out wide’). A full lower extremity MRI and a detailed knee MRI were taken. Bones and most soft tissues were segmented from images, and the generic bone architecture of the MS model was morphed into the segmented bones. The output forces from the MS model were then used as an input in the FE model of the subject’s knee. During stance, LWIs failed to reduce medial peak pressures apart from Insole 10° during the second peak. Toe in reduced peak pressures by −11% during the first peak but increased them by 12% during the second. Toe out wide reduced peak pressures by −15% during the first and increased them by 7% during the second. The results show that the work flow can assess the effect of interventions on an individual level. In the future, this method can be applied to patients with KOA.

## Introduction

With an incidence of 240 per 100 000 person-years^1^, knee osteoarthritis (KOA) is one of the largest burdens to healthcare. It is estimated that over half of adults in the US diagnosed with KOA undergo total knee replacement surgery^2^. Invasive treatment options such as osteochondral graft transplantation and total knee arthroplasty are costly procedures that are resorted to when non-invasive options such as non-pharmacological or pharmacological therapy have already been exhausted^3,4^. Currently, repairing the degenerated articular cartilage is not deemed possible, which is why alternative ways to preserve the cartilage are sought.

Knee osteoarthritis (KOA) has been shown to develop most often in the medial tibial plateau^5–7^. As excessive loading is believed to be a major contributor to the development and progression of KOA, several methods to reduce loading in the medial tibial plateau have been proposed. Gait modifications, such as toe in, toe out wide, medial knees, wide stance, and trunk sway are non-invasive techniques aimed to reduce the adduction moment in the knee, resulting in decreased medial tibial plateau loads^8–10^. However, studies have showed that a reduction in knee adduction moment (KAM) does not directly reduce stresses in medial tibial compartment^11,12^. Walter *et al*.^11^ speculate that the focus of KAM might not give a complete picture of medial knee loading. Lateral wedge insoles (LWIs) introduce a wedge in the hopes of shifting the weight from medial tibial compartment to the lateral side. The effect of lateral wedge insoles is debatable. Some studies showed a 5–7% reduction in knee adduction moment^13–15^, while a meta-analysis concluded that despite a statistically significant association between the use of insoles and reduced pain in medial KOA, the findings did not support the use of LWIs as a conservative treatment option^16^.

Direct measurement of stresses in the subject’s knee is not ethically viable, therefore computational modelling, such as *musculoskeletal* (MS), *finite element* (FE) or discrete element (DE)^17^ modelling are needed. MS models are used to investigate how the human body internally reacts to external forces and movements by providing methods to quantify forces in muscles, ligaments, and joint contact while avoiding invasive procedures. KOA patients tend to adjust their gait to alleviate pain and, in turn, alter their lower limb kinematics, kinetics, and muscle activities^18^. Since KOA patients deviate from the generalized MS model, there is a great need for individualization with regard to gait and patient’s anatomy. The Twente Lower Extremity Model 2.0 (TLEM 2.0) was implemented in the MS software package the AnyBody Modeling System (AMS) to use in combination with novel image-based morphing techniques^19^. In order for a MS model to obtain subject-specific architecture, geometric morphing techniques are applied to the TLEM 2.0 cadaver-based model to scale the bones, joints, and muscle attachments relative to the subject. Advancements in these techniques are explained in greater detail in the literature^20–24^. MS models have shown their potential at studying gait modification techniques to find the best modification for the individual^25–32^.

While MS models can estimate cartilage pressure patterns, they do not provide detailed information about the inner stresses, changes in fluid pressures, or fibril strains of cartilage. For that purpose, finite element analysis (FEA) is needed. In FEA, tissues are divided into small (‘*finite*’), discrete elements that are then assigned specific material properties, respective to the tissues they describe. Material models of the cartilage have been developed and validated against experimental results in laboratory conditions^33–35^. By implementing the forces, moments, rotations and/or translations provided by MS models as boundary conditions, FEA enables the non-invasive estimation of stresses and strains present in the cartilage during daily activities, such as gait^36^.

A multiscale model combining the use of subject-specific MS modelling and FEA of the subject’s knee joint, reflecting bones, menisci, ligaments, and tendons has yet to be created. Studies that have combined MS and FEA have either excluded patient-specific muscle attachments^37–40^, are done with cadavers only^40^, have not used subject-specific MS output data^9,38,17^, or have excluded soft tissue structures^41^.

The present study presents a method that allows scientists to simultaneously estimate net joint loads (resulting from subject-specific knee joint kinematics, external loads and ligament/muscle forces) and stresses in soft tissues of the knee during normal and modified gait. We postulated that promising techniques, such as the toe-in modification, would reduce forces in the medial compartment of the knee and furthermore reduce stresses in the medial tibial cartilage.

## Methods

**Figure 1:**
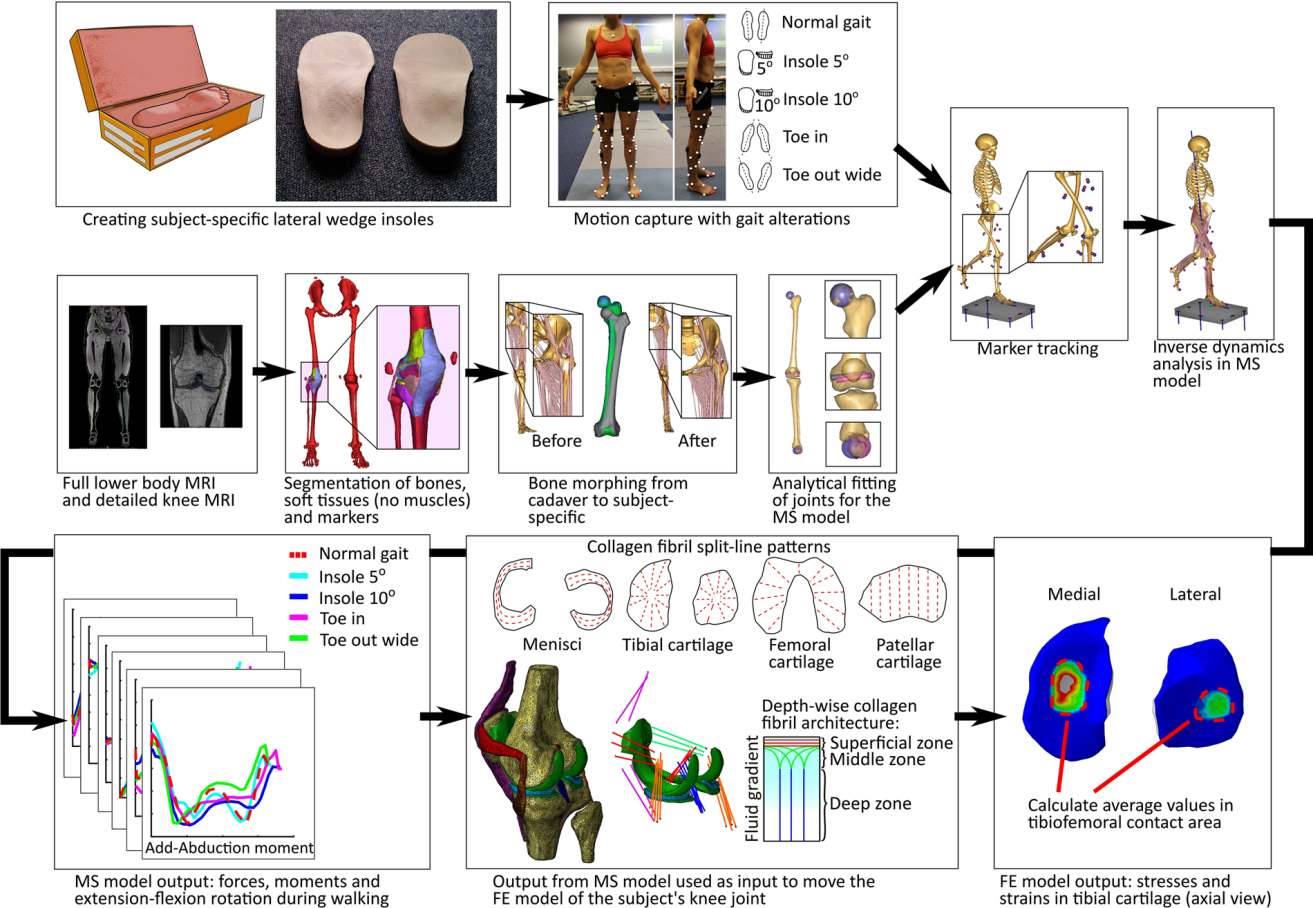
Study workflow.

Workflow of the study is presented in Fig. 1.

### Experimental data

All experiments were approved by the West of Scotland Research Ethics Service ethical committee of the NHS-Greater Glasgow and Clyde. The study was carried out in accordance with the relevant guidelines and regulations, and an informed consent was obtained from the subject. Impressions of the subject’s (27 year-old female, 172 cm, m = 61.2 kg) feet were obtained using Podotech Foot Impression Boxes (A. Algeo Ltd, Liverpool, United Kingdom). The impressions were scanned using Sense 3D Scanner (3D Systems, Rock Hill, SC, US) and transferred to Rhinoceros 3D V5 software (Robert McNeel & Associate, Barcelona, Spain) which was used for designing two sets of LWIs with 5° and 10° inclines. The orthotics were fabricated using an Airwolf 3D HDX 3D printing system (Airwolf 3D printers, Costa Mesa, USA), utilizing a soft polylactide thermoplastic to achieve necessary flexibility for LWI.

Various gait modifications were recorded at the Human Performance Lab of Glasgow Caledonian University (GCU, Glasgow, UK) using Qualisys Oqus (Qualisys Motion Capture System, Gothenburg, Sweden) camera system. 54 reflective skin markers were applied to the subject’s lower limbs at key anatomical landmarks to track lower limb motion. In addition, six markers were placed on the pelvis region and six on the torso and head to track upper body motion. A static trial of the subject standing on the force platform (Kistler 9286BA, Kistler Group, Winterhur, Switzerland) was also recorded. The subject walked with self-selected speed having their right foot come in contact with the force plate for each trial. Five trials were completed for each of the following gait styles: Normal walking (‘*Normal gait*’), toes turned slightly inward (‘*Toe in*’), toes slightly outward and a widened stance (‘*Toe out wide*’), walking with 5° lateral wedge insole (‘*Insole 5*°’), and with 10° wedge (‘*Insole 10*°’). The subject wore neutral walking shoes during all trials. The force data were synchronized with the motion capture trajectories and exported as a C3D files to be utilized by AMS.

The subject’s right knee was imaged on a 1.5 T General Electric Discovery MRI scanner using a quad knee coil. A modified Osteoarthritis Initiative (OAI) protocol^42^ based on GE scanner recommendations^43^ was applied (SAG FSPGR 3D FS and COR SPGR 3D acquisitions, slice thickness = 1 mm) to clearly distinguish between bone, articular cartilage, menisci, and ligament attachment regions. This acquisition series was labeled *Detailed Knee*. Subsequently, the subject’s lower extremities were imaged in three parts using a 3 T Siemens Prisma MRI (COR T1W-Vibe-Dixon, slice thickness = 1.4 mm) and Peripheral Angio 36 coil. During the scans, the feet were positioned in 0 degrees dorsiflexion. Prior to the scan, non-magnetic markers were applied to the subject’s pelvis and lower limbs at key anatomical landmarks coinciding with the motion capture marker locations. These three acquisitions were then stitched together by the radiologist using custom Siemens software and exported as Digital Imaging and Communications in Medicine (DICOM) files. This series of images was labeled *Lower Limbs*.

### Musculoskeletal model

The subject-specific MS model was created in the AnyBody Modeling System (AMS v 6.1 beta, AnyBody Technology, Aalborg, Denmark) using the generic human body model, excluding arms, within the AnyBody Managed Model Repository (AMMR) version 1.6. The lower extremities were updated to incorporate the TLEM 2.0 dataset.

Various anatomical structures (bone, articular cartilage, menisci, and ligaments) were required for both MS and FE models. Segmentation of these 3D geometrical surfaces from two stacks (*Detailed Knee* and *Lower Limbs*) of MRI images was completed in Mimics Research 18.0 (Materialise, Leuven, Belgium) and exported as stereolithography files (STLs). The femur, tibia, and patella were segmented from both image series (bilaterally for *Lower Limbs*). While in the *Detailed Knee*, the menisci, articular cartilage, and ligament origin and insertions were additionally segmented. In the *Lower Limbs* series, the non-magnetic markers, pelvis, fibula, talus, and foot bones on both limbs were segmented. Using functions in Mimics’ Align toolbox, the STLs from the *Lower Limb* series were registered onto the *Detailed Knee* scan to obtain a common coordinate system (MRI CS).

The hip joint was modelled as a spherical joint and the joint center estimated by fitting a sphere to the articular surface. The talocrural and subtalar joints were modelled as revolute joints and defined by fitting spheres to the articulating surfaces from the *Lower Limb* STLs^44^. Two types of joint models were implemented for the tibiofemoral (TF) and patellofemoral (PF) joints:

1. Hinge joints for both the TF and PF with one degree of freedom each (DOF)^45^.
2. Force-dependent kinematics (FDK) model with 11 DOF (6 in the TF joint and 5 DOF in the PF joint)^45^.

In both models the patella tendon was assumed rigid and its length estimated from the *Detailed Knee* MRI. For the hinge joint, the TF joint was defined with an origin at the midpoint between the centers of two circles fitted in the sagittal plane of the medial and lateral condylar articular surfaces^24^. A similar process was employed for the PF joint, selecting contact areas from the trochlear articular surface.

The FDK model was defined similarly to^45^ with an elastic foundation contact model (with a pressure modulus of 9.3 GNm^−3^) between the articular cartilages of the femur and tibia for the TF joint and the femur and patella for the PF model, respectively. The ligaments were modelled as nonlinear elastic elements with slack, toe and linear elastic regions. The toe region was set to 3% similar to^45^ and the pre-strains and ligament stiffness were defined to be identical to the FEA model (see the section about ligaments below).

Due to its lower computational cost, the hinge knee model was used for two analysis: selecting the most representative gait trials out of a total of 30 trials (Please see supplementary figure A), and investigation of the effect of weakened knee flexion and extensor muscle strength on the predicted knee reaction forces. The evaluation of gait alterations was conducted with the FDK model.

### Geometric morphing and scaling

In order to morph the femur, tibia, patella, and talus of each leg and the pelvis, we ensured the subject-specific bones had the same number of vertices as the TLEM bones (*source bones*). This was accomplished by registering a duplicated source bone to the SS bone’s coordinate system, which is defined based on the moment of inertia. The shape of vertices of the registered *source bone* were then morphed using an advanced morphing function developed by Materialise to best represent the geometry of the SS bone^46^. This morphed bone was then labelled as the *target bone* for use in AMS to scale muscle attachment sites^19^. For each bone, a modified interpolation scheme, outlined in Marra *et al*.^45^, was followed: First, an affine transformation was performed to roughly scale and register the *source bone* vertices to the respective *target bone* vertices, secondly, a tri-harmonic radial basis function (RBF) interpolation based upon the vertices of the affine-transformed *source bone* and *target bone* was performed, and third, a reverse rigid-body transformation based upon the points of the affine-transformed and RBF scaled points and the unscaled (pre affine transformation) *source bone* to bring the morphed bone back from the MRI CS to the AMS CS. The remaining 25 bones of each TLEM 2.0 foot were morphed as one rigid body using an affine transformation based on 36 anatomical landmarks capturing the shape and size of the subject-specific foot (For details, see Supplementary figure B).

To determine the location of the cluster markers relative to the morphed TLEM 2.0 model, the standing reference trial was used. A nonlinear least-square optimization problem was defined to compute the pose of the model segments based on the non-magnetic MRI markers and the corresponding markers in the motion capture data^47^. Subsequently, the local coordinates of the cluster markers were computed in the TLEM 2.0 bone coordinate systems and saved for later use.

### Muscle modelling

The muscles were modelled with a Hill-type muscle model based on the TLEM data set and scaled using a length-mass-fat scaling law^48^. The muscle recruitment problem we solved was a 3rd order polynomial cost function (*G*) with the muscle volume, *v*_*i*_, as the normalization factor and subject to the dynamic equilibrium equations and an inequality constraint ensuring that muscles can only pull and not push. Except the addition of the muscle volume normalization factor, the muscle recruitment was formulated as described by Damsgaard^49^.

1 $$\begin{array}{rrrr}\mathop{{\rm{minimize}}}\limits_{{\bf{f}}} & G({{\bf{f}}}^{(M)}) & = & \sum _{i\mathrm{=1}}^{{n}^{(M)}}{v}_{i}{(\frac{{f}_{i}^{(M)}}{{N}_{i}^{(M)}})}^{3}\\ {\rm{subject}}\,{\rm{to}} & {\bf{C}}{\bf{f}} & = & {\bf{d}}\\  & 0\le {{\bf{f}}}_{i}^{(M)},\,\,i & = & \mathrm{1,}\cdots ,{n}^{(M)}\end{array}$$

where **f** ^(*M*)^ is a vector consisting of *n* ^(*M*)^ unknown muscle forces, **C** is the coefficient matrix for the dynamic equilibrium equations, **f** is made up of all unknown forces in the problem, which depends on whether the knee is modelled as a hinge or with FDK (see the section below). ${f}_{i}^{(M)}$ denotes the *i th* muscle force, and *N*_*i*_ *N*_*i*_ is the muscle strength of the *i th* muscle. **d** = [**d**_1_ *T*…**d**_*n*_]*T* d = [d_1_ *T*…d] T*n*contains the external loads and inertia forces:

2 $${{\bf{d}}}_{j}={{\bf{g}}}_{j}^{({\rm{app}})}-[\begin{array}{cc}{m}_{j}{\bf{I}} & {\bf{0}}\\ {\bf{0}} & {{\bf{J}}}_{j}^{\text{'}}\end{array}]{\dot{{\bf{v}}}}_{j}-[\begin{array}{c}{\bf{0}}\\ {\tilde{\omega }}_{j}^{\text{'}}{{\bf{J}}}_{j}^{\text{'}}{\omega }_{j}^{\text{'}}\end{array}]$$

${{\bf{g}}}_{j}^{(app)}$ contain the forces and moments applied around the center of mass of the *j th* segment in body-fixed coordinates. *m*_*j*_, **J**′*j*, ${\dot{{\bf{v}}}}_{j}$ and ${\omega }_{j}^{\text{'}}$ are the mass, mass moment of inertia in body-fixed coordinates, and the linear and angular velocity of the *j th* segment. The tilde indicates the skew-symmetric matrix.

Within the TLEM 2.0 data set, muscles are sub-divided to account for wide origin/insertion areas. Without a normalization factor, this sub-division affects the estimated muscle and joint reaction forces as shown by Holmberg and Klarbring^50^. Therefore, a normalization factor based on muscle volume was introduced as similarly done in previous studies^45,51^.

### Marker kinematics, inverse dynamics, Force-dependent Kinematics and output for FE model

The subject-specific model was run using an inverse kinematic technique^47^, tracking the motion capture marker trajectories for each of gait trials. In this process, the TF and PF joints were modelled as hinges. The resulting translation and rotation of pelvis and the joint angles, angular velocities, angular accelerations and the measured ground reaction forces and moments (GRF & Ms) were then used as input for the inverse dynamic analysis or FDK simulations similar to Marra *et al*.^45^ depending on whether the hinge or FDK knee model was applied.

In these two cases, the vector of unknown forces, **f**, and corresponding coefficient matrix, **C**, in equation (1) were set up differently. When a hinge knee was applied, ${\bf{f}}={[{{\bf{f}}}^{(M)T}{{\bf{f}}}^{(R)T}]}^{T}$, where **f** ^(*R*)^ are the unknown joint reaction forces. The coefficient matrix **C **= [**C** ^(*M*)^ **C** ^(*R*)^] is made up of the coefficient matrix for the muscles, **C** ^(*M*)^, and for the reaction forces **C** ^(*R*)^. These coefficient matrices can be determined by the derivative of the origin to insertion length and the joint constraint equations with respect to a set of coordinates that correspond to the linear and angular velocity of the segments as shown by Damsgaard *et al*.^49^.

When performing FDK analysis, specific DOFs of the model are identified, where the movement is computed for each time step based on an assumption of quasi-static force equilibrium (see Andersen.. and Marra *et al*.^45^). In the FDK knee model, these are all the DOFs of the knee except TF and PF flexion. All other joint angles and the TF flexion angle obtained from the inverse kinematic analysis and the PF flexion is controlled by an assumption of a rigid patellar tendon. To accomplish this type of analysis, an iterative algorithm is applied to determine the position and orientation at each time step of these FDK DOFs such that static equilibrium along these is obtained. To this end, residual forces along these FDK DOFs are introduced among the unknown forces, **f** = [**f** ^(*M*)T^ **f** ^(*R*)T^ **f** ^(*FDK*)T^]^T^ and with coefficient matrix **C** = [**C** ^(*M*)^ **C** ^(*R*^ **C** ^(*FDK*)^] and the numerical solver searches for the positions of the FDK DOFs until the residual forces are below a specified tolerance of 0.3 N (Nm) similarly to Marra *et al*.^45^. For full details of FDK analysis, we refer the reader to Andersen *et al*.^52^.

To transfer the TF extension-flexion knee movement and estimated forces and moments affecting on the patella and femur of the MS model to the FEA model, identical reference frames were defined in both models based on the MRI scans. For the right femur, tibia and patella, a coordinate system was defined with the x-axis pointing from the lateral to the medial epicondyle, the y-axis orthogonal to the x-axis and pointing towards the hip joint center and the z-axis the cross product of the two. The origin was located midway between the epicondyles. This coordinate system was transformed to the morphed TLEM2.0 right femur, tibia, and patella bones and moved rigidly with the respective bone. These we denote the *FEA coordinate systems*. The origins of the FEA coordinate systems of femur and patella, we denote the *femoral reference point* and the *patellar reference point*, respectively.

The extension-flexion angle was measured as the first angle (abduction-adduction and internal-external rotation were second and third respectively) in a Cardan angle representation from the femur to the tibia. A free body diagram was set up for the right femur which sums up all forces and moments from the muscle forces, hip joint reaction forces, gravity, and inertia forces but with the reaction forces of the tibiofemoral joint and the patellofemoral joint omitted. The equivalent force and moment of these were computed around the *femoral reference point* but represented in the basis of the tibia *FEA coordinate system* as this is used as the global reference frame in the FEA model as shall be clear later. A similar procedure was applied to compute the equivalent force and moment for patella, where the reaction forces of the patellofemoral joint and the reaction forces in the rigid patellar tendon were excluded. These forces and moments were computed around the *femoral reference point* and given in the basis of the tibia *FEA coordinate system*.

**Figure 2:**
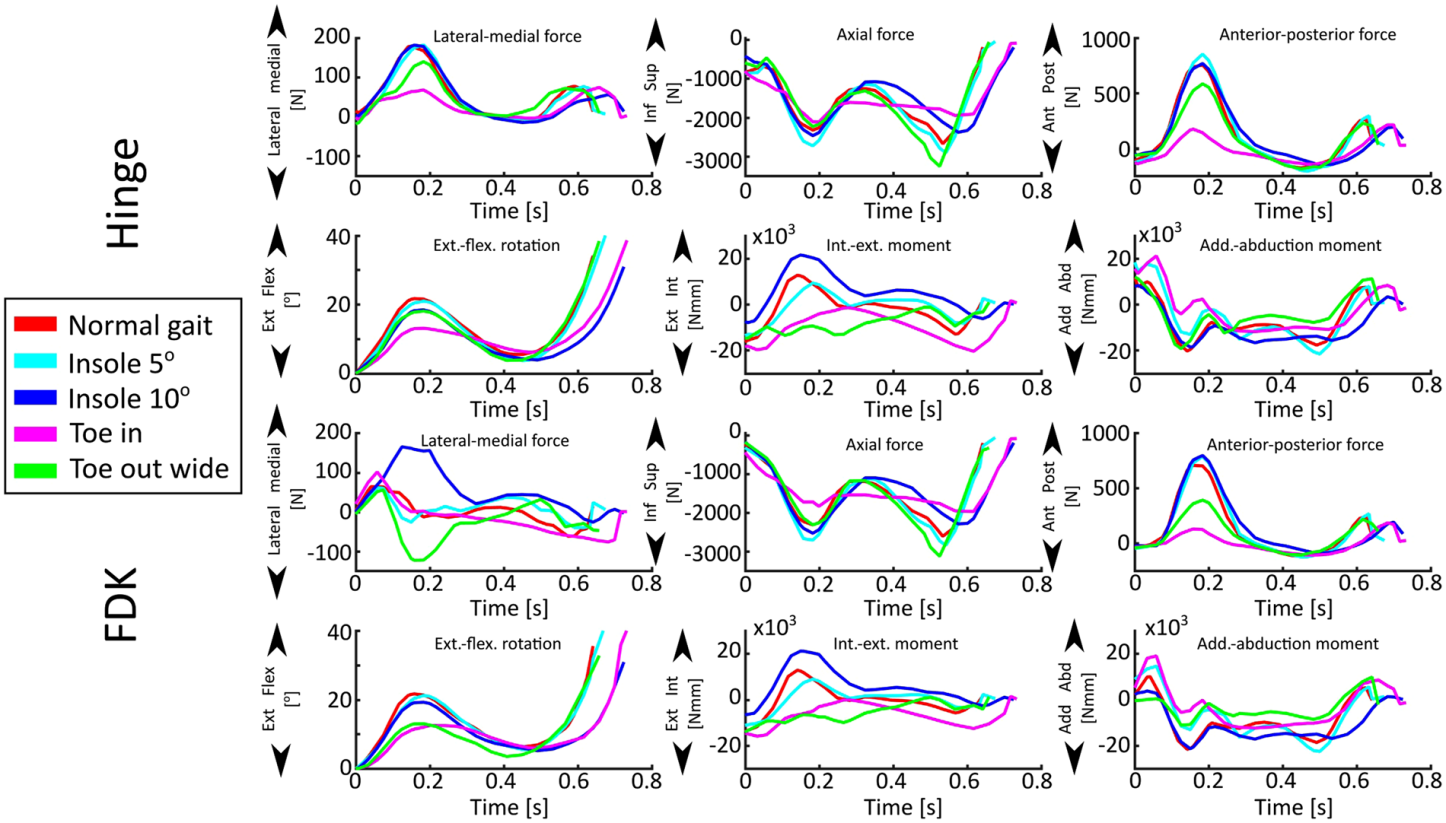
Musculoskeletal model outputs for femur (**top**: Hinge knee model, **bottom**: FDK knee model), used as the input for the finite element model of subject’s knee joint. Note: patellar inputs omitted for clarity.

The MS model provided five inputs for the FE model (Fig. 2) of each walking technique (*Normal gait*, *Toe in*, *Toe out wide*, *Medial knees*, *Insole 5*°, and *Insole 10*°), so 30 in total. Out of each technique, three trials with a stance phase time most comparable with normal walking (for this subject, approximately 0.65 s) were selected (*i.e*. total of 18). This was done because slowing the walking pace reduces the impact loads on cartilage, and would, therefore, possibly show artificially decreased stresses in the cartilage. For the same reason, all trials of the medial knees technique had to be excluded due to a 35% longer stance phase compared with normal walking, leaving a total of 15. For the remaining techniques, each with three trials, the trial with the most average trend was selected as the final input for the FE model, resulting in a total of 5 inputs. Please see Supplementary figure B for a plot of internal-external and adduction-abduction moments for all trials.

### Finite element model

The soft tissues were segmented from the *Detailed Knee* MRI acquisition, exported as STL files and then transformed into solid geometry form using a custom Matlab R2015b script (Mathworks Inc., Natick, MA, United States). The solid geometries were imported into Abaqus (v. 6.13–3, Dassault Systémes Simulia, Vélizy-Villacoublay, France). Tibial cartilage was kept at the MRI position, while all other tissue geometries were moved respective to tibia, from their MRI positions into the position at the beginning of stance phase. The cartilages and menisci were meshed using 8-node porous elements (element type = C3D8P). The total number of elements for femoral, tibial and patellar cartilages was 7245, 7830 and 3075, respectively. For the lateral and medial meniscus, the number of elements was 8160 and 5250, respectively.

### Material model

A fibril-reinforced poroviscoelastic (FRPVE) material model^33–35^, was used for all cartilages and menisci, while bones were defined rigid. In the FRPVE material model, the cartilage is considered a biphasic material consisting of a fluid phase and a solid matrix. The solid matrix consists of a fibrillar part, mimicking the collagen fibril network of articular cartilage, and a Neo-Hookean poro-hyperelastic part, mimicking the proteoglycans, chondrocytes and smaller constituents. The total stress *ρ* ^*tot*^ is given as

3 $${\rho }^{tot}={\rho }^{f}+{\rho }^{nf}-{{\rm{I}}}_{{\bf{P}}}$$

where *ρ* ^*f*^ is the fibrillar stress, *ρ* ^*nf*^ non-fibrillar stress, **p** fluid pressure and **I** unity tensor. Further, the fibrillar stress *ρ* ^*f*^ is defined as a sum of stresses in each individual collagen fibril:

4 $${\rho }^{f}=\sum _{{\bf{i}}=1}^{N}{\rho }_{i}^{f}$$

where ${\rho }_{i}^{f}$ is the stress of one fibril and N the total number of fibrils. The viscoelastic behaviour of collagen fibrils is modelled using a linear spring (spring coefficient *E*_*0*_) in parallel with a set of a non-linear spring (spring coefficient *E*_*ε*_) in series with a damper (damping coefficient *η*). The collagen fibrils consist of primary and secondary fibrils, with their relative density denoted by *D*. Primary fibrils are oriented parallel to the cartilage surface in the superficial zone, randomly oriented in the middle zone and perpendicular to the surface in the deep zone. In addition, the macro-pattern of primary collagen fibrils in the superficial zone follows the split-line pattern of cartilages^53–55^. The secondary fibrils are randomly oriented. Similarly to the cartilages, the menisci were also modelled as biphasic material using the FRPVE material model. However, in the menisci, the primary collagen fibrils are oriented circumferentially. The full list of material parameters in cartilages and menisci are given in Table 1. For more detailed description of the implementation of the material model, please see these previous studies^56–59^.

**Table 1:** Material parameters implemented for cartilages and menisci.

Material properties	Femoral cartilage	Tibial cartilage	Patellar cartilage	Menisci
*E*_*m*_ (MPa)	0.215^33^	0.106^33^	0.505^33^	0.5^89^
*v*_*m*_	0.15^34^	0.15^34^	0.15^34^	0.36^89^
*E*_0_ (MPa)	0.92^33^	0.18^33^	1.88^33^	28^89^
*E*_*ε*_ (MPa)	150^33^	23.6^33^	597^33^	—
*k0* (×10^15^m^4^/Ns))	6^33^	18^33^	1.9^33^	1.25^90^
*M*	5.09^33^	15.64^33^	15.93^33^	5.09^33^
*η* (MPa s)	1062^33^	1062^33^	1062^34^	—
*n*_*f*_	0.8–0.15_*Z*_^91^	0.8–0.15 *z* ^91^	0.8–0.15_z_^91^	0.72^90^
*D*	12.16^33^	12.16^33^	12.16^33^	12.16^33^

*E*_*m*_ = Non-fibrillar matrix modulus, *v*_*m*_ = Poisson’s ratio, *E*_0_ = initial fibril network modulus, *E*_*ε*_ = strain-dependent fibril network modulus, *k*_0_ = initial permeability, *M* = exponential term for the strain-dependent permeability, *η* = damper coefficient, *n*_*f*_ = fluid fraction as a function of the cartilage depth (*z*), *D* = ratio of primary collagen fibrils to secondary fibrils.

### Ligaments

The ligaments were defined as linear spring elements (SPRINGA). The ligament stiffnesses, defined as spring coefficients, were obtained from Marra *et al*.^45^: anterior cruciate ligament (ACL) 306 Nmm^−1^, posterior cruciate ligament (PCL) 406 Nmm^−1^, lateral collateral ligament (LCL) 99 Nmm^−1^, medial collateral ligament (MCL) 168 Nmm^−1^, lateral patellofemoral ligament (LPFL) 68 Nmm^−1^, and medial patellofemoral ligament (MPFL) 49 Nmm^−1^. Pre-strains for ACL, PCL, MCL, LCL, MPFL, and LPFL, were defined as 3%, 3%, 2%, 3%, 5% and 5%, respectively, by modelling tests from radiographic knee laxity studies^60,61^. The patellar tendon was also defined as a linear spring element with a spring coefficient of 545 Nmm^−1^ without prestrain^62^. No quadriceps tendon was defined as an explicit structure as the forces and moments that were obtained from the MS model account for the forces in the quadriceps.

### Contact definitions and boundary conditions

In the FE model, the tibial cartilage was fixed at the cartilage-bone interface. During simulations, all forces, moments and extension-flexion rotation were applied to femur and patella with respect to the tibia *FEA coordinate system*. Surface-to-surface contact was used for all contacting surfaces, the master surface being a surface and the slave surface a node surface. Patellar cartilage was defined as a master to femoral cartilage, femoral cartilage master to tibial cartilage and menisci, and tibial cartilage master to the menisci. Bottom elements of the menisci were subdivided into smaller elements and tied to the upper meniscal elements using a constraint in order to improve convergence in the tibia-meniscus contact.

In the simulations, first the cartilages and menisci were brought into light contact with each other and ligament pre-strains were applied. Next, the forces and moments present at the start of stance phase of gait were applied. Finally, the stance phase of gait was simulated by applying translational forces (anterior-posterior, axial, and medial-lateral), moments (internal-external, varus-valgus) and a rotation (extension-flexion) to the *femoral reference point*. This point was tied to the cartilage-bone-interface of femoral cartilage as well as all the femoral ligament attachments^63^. Similarly, patellar forces and moments were applied through the *patellar reference point*. The *patellar reference point* was tied to the cartilage-bone interface of patellar cartilage and was initially located at the same place as *femoral reference point*, up until the application of femoral and patellar forces and moments. During gait, all forces and moments acting on the patella were applied through the *patellar reference point*.

### Analysis of results

First, the effect of knee flexor and extensor strength on tibial reaction forces was studied with the hinge MS model. After this, all results were obtained using the FDK knee joint. Peak contact pressures were determined in the tibial cartilage surface. In addition, average contact pressures, maximum principal stresses, maximum principal strains, fibril strains, and fluid pressures were analysed at the tibial cartilage surface. Tibiofemoral contact area was defined by choosing tibial surface nodes with a contact pressure exceeding 0.01 MPa. Additionally, the sum of reaction forces at the bottom of medial and lateral tibial cartilages were calculated.

## Results

### Knee flexor and extensor muscle strength

**Figure 3:**
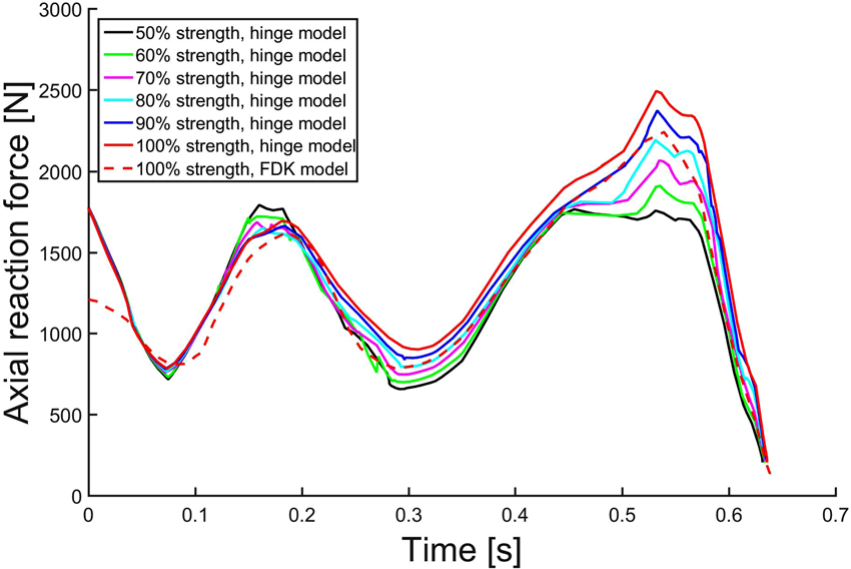
Effect of flexor and extensor strength on tibial reaction forces. Hinge knee (in the MS model) extensor strength scaled from 50% to 100% (normal). Force-dependent kinematics (FDK) model with normal gait in dashed red line.

When varying the knee flexor and extensor muscle strength in the hinge knee joint model from 100% (normal strength) to 50% (severely weakened), a difference of 720 N was seen during the second axial peak force (Fig. 3). Normal gait with the FDK knee decreased peak reaction forces compared with the hinge model (2239 N vs. 2492 N, respectively).

### Cartilage deformation

**Figure 4:**
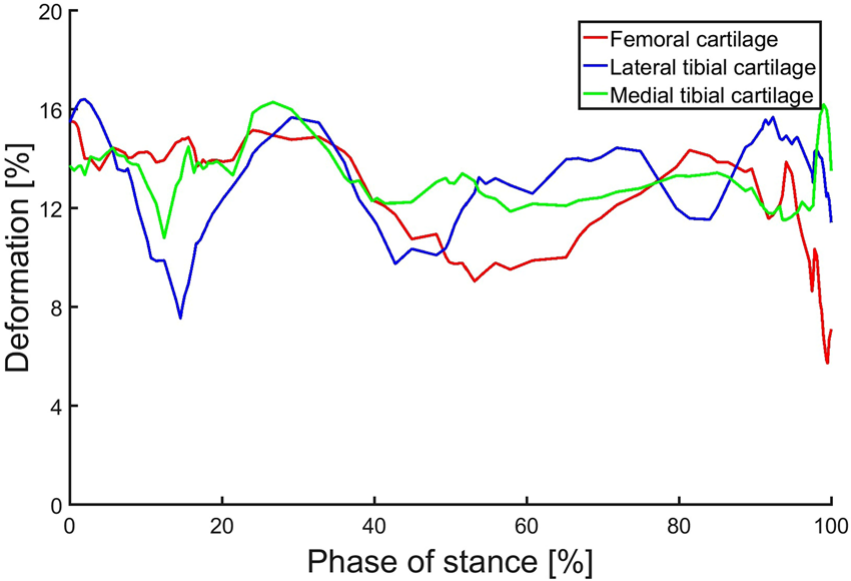
Maximum cartilage deformations during the stance phase of gait, compared with resting cartilage from MRI.

In the tibiofemoral contact, maximum cartilage deformation (compared with resting cartilage thickness from MRI) for femoral cartilage was 16% during the stance phase of gait (Fig. 4). The maximum medial and tibial cartilage deformations was also 16% and 16%, respectively.

### Peak contact pressures

**Figure 5:**
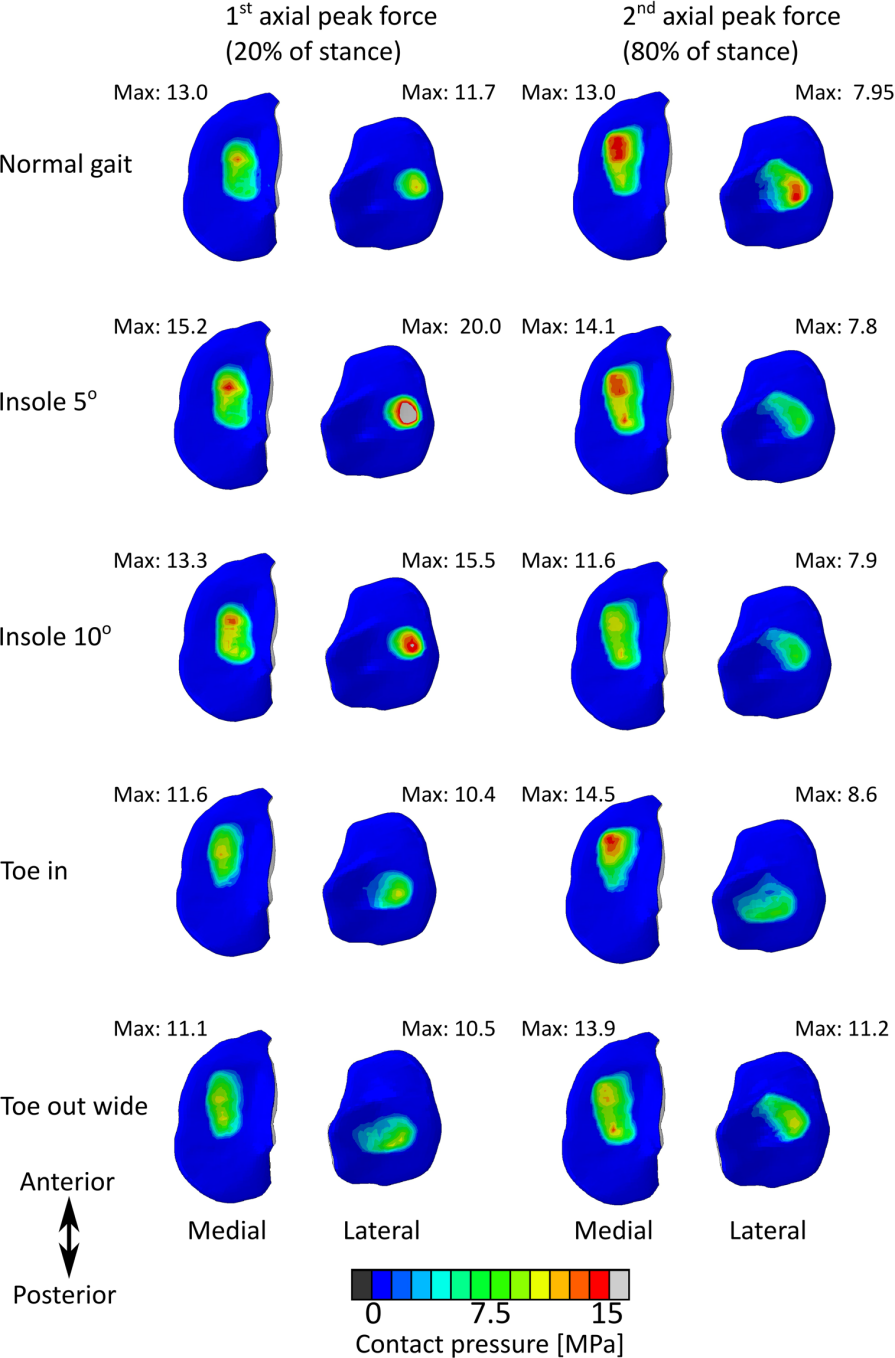
Results of FE analysis: Contact pressures in the tibial cartilage surface during the first and second axial peak forces (20% and 80% of stance). FDK model.

Figure 5 shows the peak contact pressures during the first and second axial peak forces (20% and 80% of stance) during gait. During the first peak, Insole 5° and Insole 10° increased pressures in the medial tibial cartilage by 17% and 14%, respectively (Table 2), while Toe in and Toe out wide reduced them by −11% and −15%, respectively.

**Table 2:** Peak contact pressures in tibial cartilage surface during first and second peak axial reaction forces of stance, as observed in the FE model.

	Gait alteration				
	Normal gait	Insole 5°	Insole 10°	Toe in	Toe out wide
1^*st*^ peak, medial	13.0 MPa	15.2 MPa (+17%)	13.3 MPa (+14%)	11.6 MPa (−11%)	11.1 MPa (−15%)
1^*st*^ peak, lateral	11.7 MPa	20.0 Mpa (+71%)	15.5 Mpa (+33%)	10.4 Mpa (−11%)	10.5 Mpa (−10%)
2^*nd*^ peak, medial	13.0 MPa	14.1 MPa (+8%)	11.6 MPa (−11%)	14.5 MPa (+12%)	13.9 MPa (+7%)
2^*nd*^ peak, lateral	7.95 MPa	7.8 MPa (−2%)	7.9 MPa (−1%)	8.6 MPa (8%)	11.2 MPa (+41%)

During the second axial peak force, Insole 5° increased peak contact pressures by 8%, while Insole 10° reduced them by −11%. Toe in and Toe out wide methods increased peak contact pressures by 12% and 7%, respectively.

During the first axial peak force, increased lateral peak pressures were observed in Insole 5° and Insole 10° (71% and 33%, respectively). Toe in and Toe out wide decreased lateral peak pressures by −11% and −10%, respectively.

During the second axial peak force, similar lateral contact pressures were seen in Insole 5° and Insole 10° models, compared with the normal gait. Toe in and Toe out wide increased pressures by 8% and 41%, respectively.

### Mean stresses and strains

**Figure 6:**
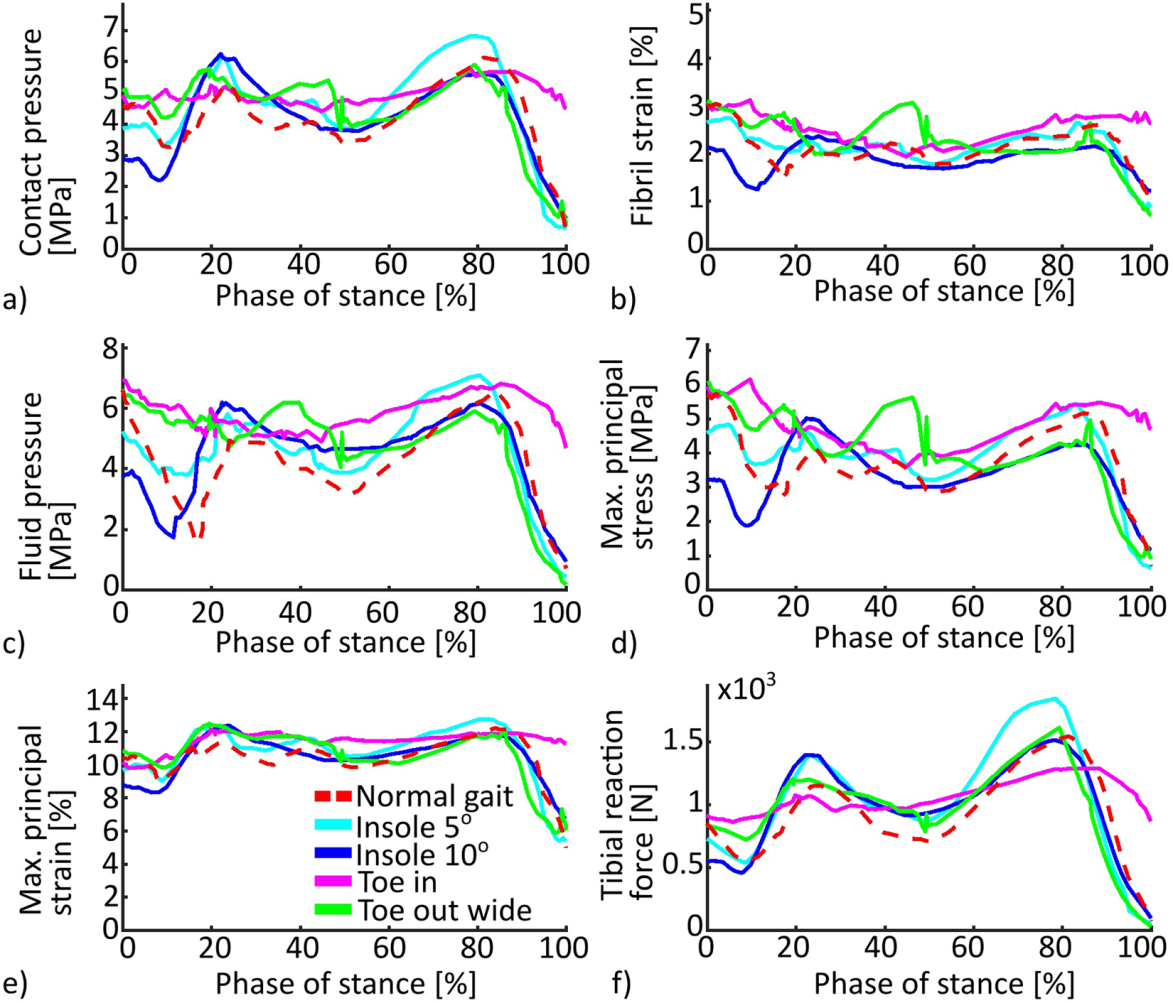
Mean values in the medial tibial cartilage surface **(a)**–**(e)** and summed axial reaction forces in the medial tibial cartilage-bone interface **(f)**.

In the medial tibial cartilage, mean contact pressures were elevated in all gait alteration models compared with the normal gait, during both axial peak forces (Fig. 6). Highest medial tibial reaction forces occurred in the Insole 5° and Insole 10° models. All models deviated from the Normal gait model most during the heel strike, especially in fluid pressure. Toe out wide showed an increase in all stresses and strains during midstance.

**Figure 7:**
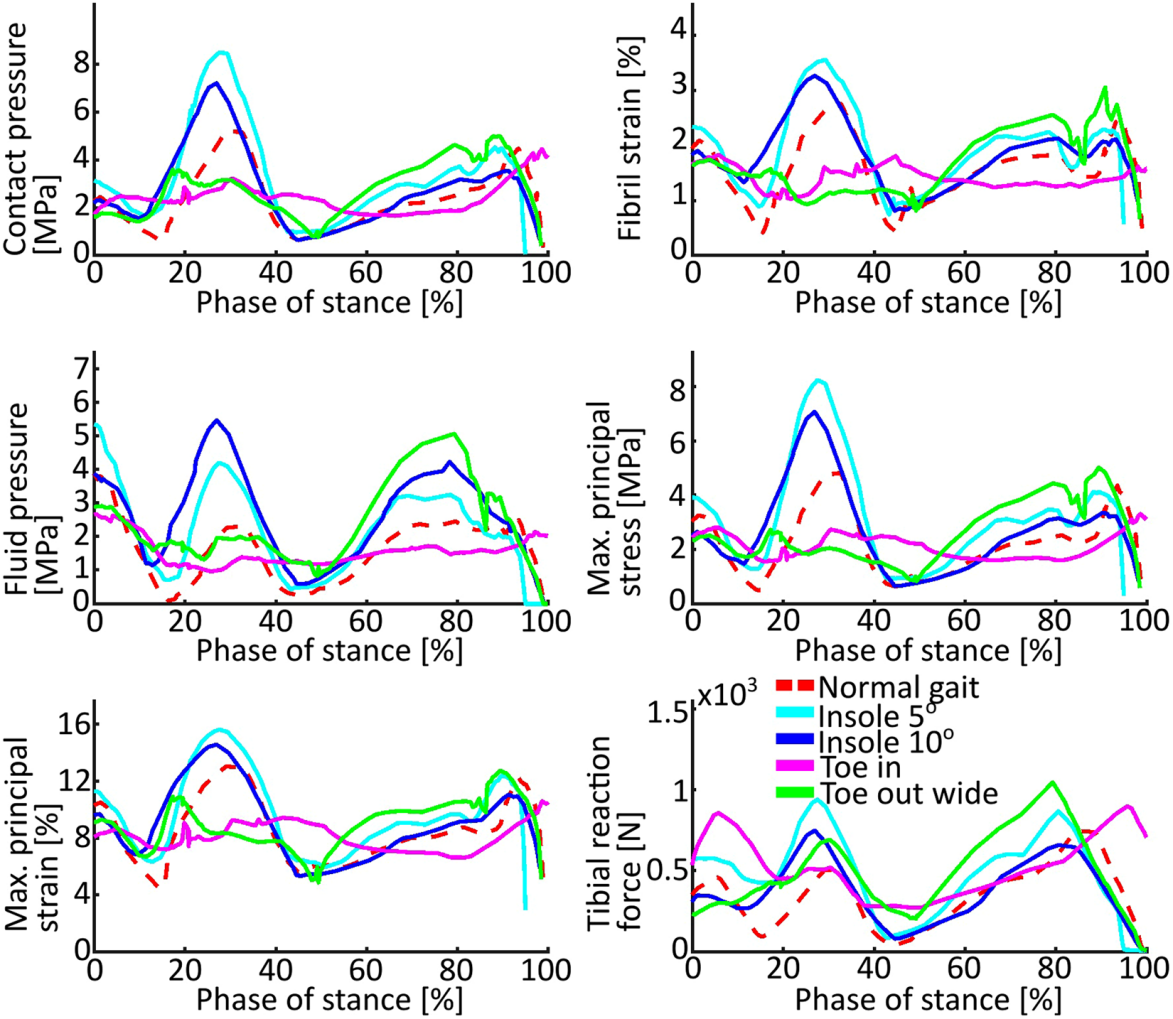
Mean values in the lateral tibial cartilage surface **(a)–(e)** and summed axial reaction forces in the lateral tibial cartilage-bone interface **(f)**.

In the lateral tibial cartilage, Insole 5° and Insole 10° substantially increased mean stresses and strains during the first axial peak force of stance (Fig. 7). Toe in showed a similar trend to normal gait, showing decreased values during the first axial peak.

## Discussion

We have combined MS and FE modelling with a highly subject-specific model creation in order to establish a workflow to investigate whether gait alteration techniques reduce medial tibial cartilage stresses and strains on an individual basis. The subject’s lower extremities were imaged using a lower-limb MRI and a detailed knee MRI. Subsequently, a bone-morphing technique was applied in order to anatomically scale the generic bone architecture of the MS model to the subject, including muscle attachment sites. A hinge-joint and a FDK joint model were implemented to the knee and the subject’s gait trial data were processed into force and moment outputs. Finally, the outputs were used in the FE model to evaluate stresses and strains in the tibial cartilage surface. This method allows non-invasive evaluation of the effectiveness of gait modification techniques in terms of actual stresses in the tibial cartilage, not just the knee adduction moment.

Peak reaction forces of 3.6xBW were observed, which is higher than the 2.5–3.2xBW reported in literature^64–66^. However, we argue that because the contract force data is obtained from TKR joint implants, the forces are lower than those of a healthy person, like the subject of this study. It is known that the quadriceps and hamstring strength of OA patients is weakened^67,68^. When the normal gait was simulated with 50% of the extensor strength, compared with the normal gait, peak reaction forces of 2.8xBW were observed. This falls right into the reported range and supports our argument. Furthermore, the present MS model has shown an RMS error of <0.3 BW for the tibiofemoral contact forces during gait, when tested against *in vivo* measured tibiofemoral joint forces, courtesy of the fifth grand challenge for the 7th World Congress of Biomechanics^45^. The data was gathered from a tibial implant with force-measuring six load components transferred through the prosthesis^12,69,70^.

Peak contact pressures of 13 MPa were observed during normal gait, which is in the reported range of 12–15 MPa^71,72^. In addition, the maximum cartilage deformations were within 6–16% of resting cartilage thickness in the tibiofemoral contact, which is in the reported range of deformation during gait^73^ and below reported strain threshold of 30% for human chondrocyte apoptosis^74^.

Both the Insole 5° and Insole 10° increased peak contact pressures in the lateral side during the first axial peak reaction force, but failed to reduce medial contact pressures. This is likely due to the elevated reaction forces the subject’s medial tibia underwent when walking with insoles. This is supported by the fact that during the second axial peak force, when the medial reaction forces were similar in Insole 10° and Normal gait, medial contact pressures did indeed decrease. Furthermore, the peak lateral contact pressures were dangerously high^75^ in the Insole 5°. Many studies suggest that LWIs do not reduce KAM^76–78^, or medial tibial contact pressures^79^. On the other hand, many studies have reported a significant change in KAM^13–15,80^. Crenshaw *et al*.^80^ reported a −7% reduction in KAM, caused by LWI, and suspected the results might depend on the type of insole used. None of the studies above criticizing LWIs mention having used subject-specific, tailored insoles. In the present study, the LWIs used were tailored to the subject using 3D printing technique, yet the results still do not support the use of LWIs for this subject as an intervention.

The MS model predicted reduced axial forces in the medial tibial cartilage for Toe in method, which resulted in decreased medial tibial reaction forces. Toe in reduced KAM during the first peak force, which is in agreement with previous studies^8,81^. However, while the peak medial contact pressures decreased during the first axial peak force, they actually increased during the second peak. This result is likely due to a change in the size and location of the tibiofemoral contact area. The results suggest that for this subject, the Toe in method might not be optimal.

Toe out wide reduced KAM the most, especially during the second axial peak force, which is in accordance with previous studies suggesting that walking with toes out reduces KAM by up to 40%^82–84^. Previous studies^82–84^, indicate that the method has little effect on the first peak force during stance. In this study, the greatest reduction was actually observed during the first peak, while the peak contact pressures were slightly increased during the second peak. For this subject, the Toe out wide method yielded best results out of all methods.

All gait alteration techniques managed to reduce KAM during the stance apart from the second axial peak force, when Insole 5° slightly increased the KAM. The correlation between KAM and mean medial contact pressures varied from 0.25 to 0.80 (see Supplementary figure C), best correlation being in Insole 10° and worst in Toe in method. Normal gait had a correlation of 0.45.

It needs to be noted that the stance phase of Insole 10° and Toe in was approximately 10% longer than in the normal gait, which may have reduced the stresses. For the subject, it was difficult to maintain a consistent walking speed during the gait alterations, which is why we used a criterion of maximum 10% deviation in stance time in the trials. Stance time was chosen instead of walking speed, because the highest forces on the knee occur during the stance phase.

Due to the methodological nature of this study, the largest limitation is the use of only one subject. We want to emphasize that the focus of this study is to establish a method to study the effect of gait alterations, not provide prove or disprove a particular method. Therefore, before establishing any generalized findings, the workflow needs to be applied to extended patient cohorts. For this purpose, several OA patients with medial tibial cartilage wear will be investigated in the future.

Within the MS model, we applied revolute joint models for the tibiofemoral and patellofemoral joints when processing the 30 gait trials as well as the flexor and extensor knee muscle strength testing due to the computational requirements of a full FDK model. As the knee is a complex joint and does demonstrate significant movements besides flexion/extension^85^, this will result in some uncertainty in the muscle strength study. It should be noted, however, that we observed only minor differences when comparing the predicted knee contact forces with an FDK-based model and a hinge.

One of the advantages of applying FDK or other similar algorithms to predict secondary joint kinematics (see Brandon *et al*.^86^ for a review of methods) is that these are estimated based on joint mechanics and the dynamics of the musculoskeletal system rather than pure kinematic constraint equations. As shown by Marra *et al*.^45^ and Lenhart *et al*.^87^, these types of models are capable of predicting secondary joint kinematics that generally agree with *in vivo* measurements.

A limitation of all skin marker-based movement analysis is soft tissue artefacts^85^. As shown by Benoit *et al*.^85^, only knee extension-flexion can be accurately estimated from the skin markers. Due to the ethical and technical issues of obtaining accurate full lower extremity kinematics, no data set exists which we can use to fully validate the kinematics of our models. However, because the FDK model only relies on the estimated gross joint kinematics and predicts the secondary knee joint kinematics based on the forces, the knee kinematics and kinetics are likely better estimated than if a kinematic constraint-based model was applied. Future studies should investigate this further.

While we have implemented subject-specific geometry based on MRI, subject-specific movements based on skin markers, and subject-specific insoles, there are still parameters in the models that were obtained from literature and for which subject-specific parameters should be included in future studies. First of all, generic ligament parameters were applied in the FEA models due to the lack of experimental setups to estimate the full 3D joint laxities of the tibiofemoral and patellofemoral joints from which the ligament stiffness and slack lengths can be identified. Secondly, due to ethical reasons subject-specific material parameters cannot be currently obtained for the cartilage material model, but from cadaver studies and bovine samples. Thirdly, generic muscle-tendon parameters were applied in the models. Ideally, this should be personalized e.g. based on isometric and isokinetic measurements for instance using optimization-based approaches^88^. While these strength measurements can be performed, there is the issue of whether the subject is producing maximal forces especially in a patient population. Future work should investigate methodologies for obtaining these parameters with as few measurements as possible.

In conclusion, we propose a workflow to investigate the effect of gait alterations on cartilage stresses by combining motion capture, ground reaction forces, MR images, bone morphing, multibody dynamics, and finite element analysis. This method enables subject-specific and non-invasive evaluation of low-cost clinical interventions, aiming to unload the medial tibial cartilage. Currently, creating the models is time-consuming, especially tissue segmentation. In order to bring the method to clinical practice, improvements in automatic tissue segmentation are needed. The cartilage material may in fact be overly complex for the present study, but its features are required when implementing the method to KOA patients in order to simulate changes in the structure of OA cartilage. The presented workflow provides groundwork to develop patient-specific models and optimised treatments on a more individualised basis.

## Electronic supplementary material

Supplementary Information
