# Supplementary material for: Workflow assessing the effect of gait alterations on stresses in the medial tibial cartilage - combined musculoskeletal modelling and finite element analysis
Source: Sci Rep. 2017 Dec 12;7:17396. doi: 10.1038/s41598-017-17228-x (PMC5727195; doi:10.1038/s41598-017-17228-x)
Supplement: Supplementary file 1 — Supplementary Information [file 41598_2017_17228_MOESM1_ESM.pdf]

Workflow assessing the effect of gait alterations on stresses in the medial tibial cartilage – combined musculoskeletal modelling and finite element analysis

K S Halonen[1,*], C M Dzialo[2], M Mannisi[3], M S Venäläinen[4], M de Zee[1], and M S Andersen[2]

[1]Department of Health Science and Technology, Aalborg University, Fredrik Bajers Vej 7D, DK-9220 Aalborg, Denmark

[2]Department of Mechanical and Manufacturing Engineering, Aalborg University, Fibigerstræde 16, DK-9220 Aalborg, Denmark

[3]School of Health and Life Science, Glasgow Caledonian University, Cowcaddens Rd, G4 0BA, Glasgow, United Kingdom

[4]Department of Applied Physics, University of Eastern Finland, POB 1627, FI-70211 Kuopio, Finland

*ksh@hst.aau.dk

Supplementary Info

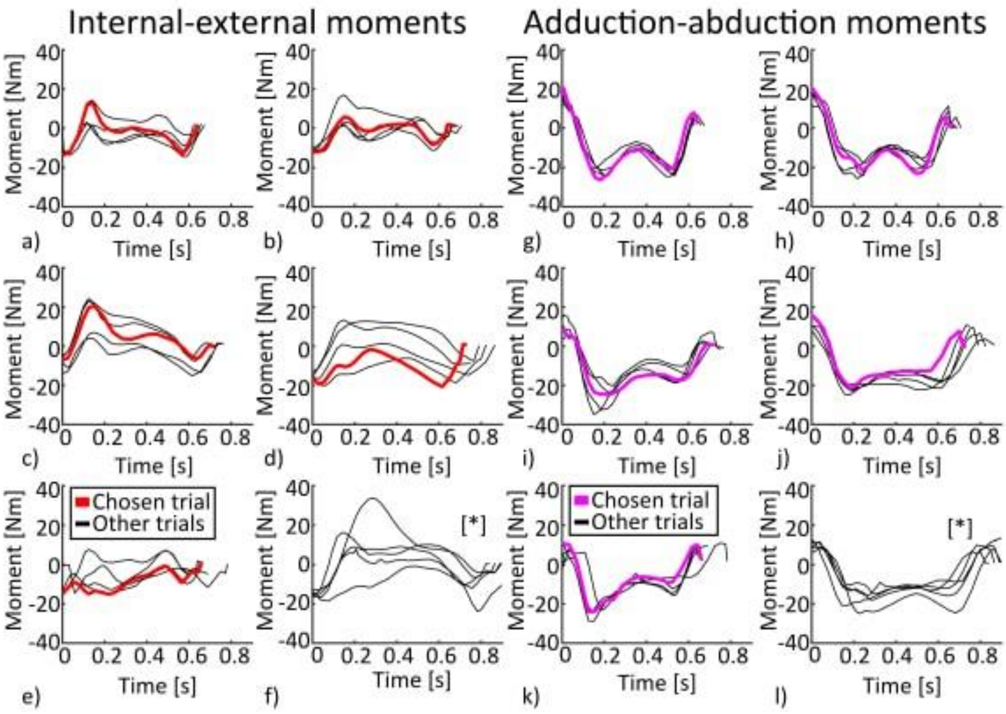

Supplementary Figure A. Left: Internal-external moments in **(a)** normal gait, **(b)** Insole 5°, **(c)** Insole 10°, **(d)** Toe in, **(e)** Toe out wide, and **(f)** Medial knees. **Right:** Adduction-abduction moments in (g) normal gait, (h) Insole 5°, **(i)** Insole 10°, **(j)** Toe in, **(k)** Toe out wide, and **(l)** Medial knees. The chosen trial for the FE input is highlighted in color. [*]: Medial knees were excluded from the analysis due to large difference in stance duration compared with normal gait.

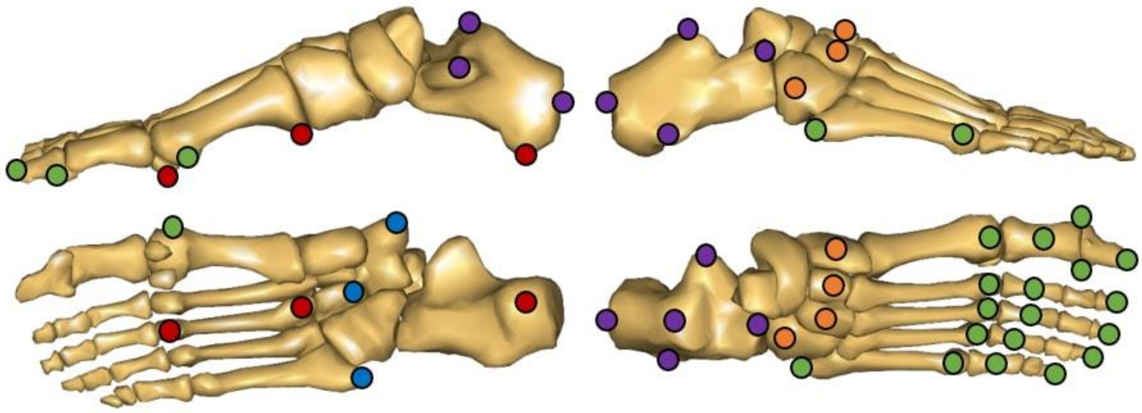

Supplementary Figure B. Anatomical landmarks on right foot: (**Red circles**) Longitudinal arch selection 1, 2, 3 (**Blue circles**) Transverse arch selection 1, 2, 3 (**Green circles**) 2nd – 5th distal and 1st – 5th proximal phallanges, 1st – $5^{\mathbf{th}}$ metatarsal heads, 5th proximal metatarsal, Hallux: distal, lateral, and medial, tuberosity navicular, (**Orange circles**) Cuboid and Cuneiform: lateral, intermediate, medial (**Purple circles**) Calcaneous: superior, anterior, posterior, lateral, and medial.

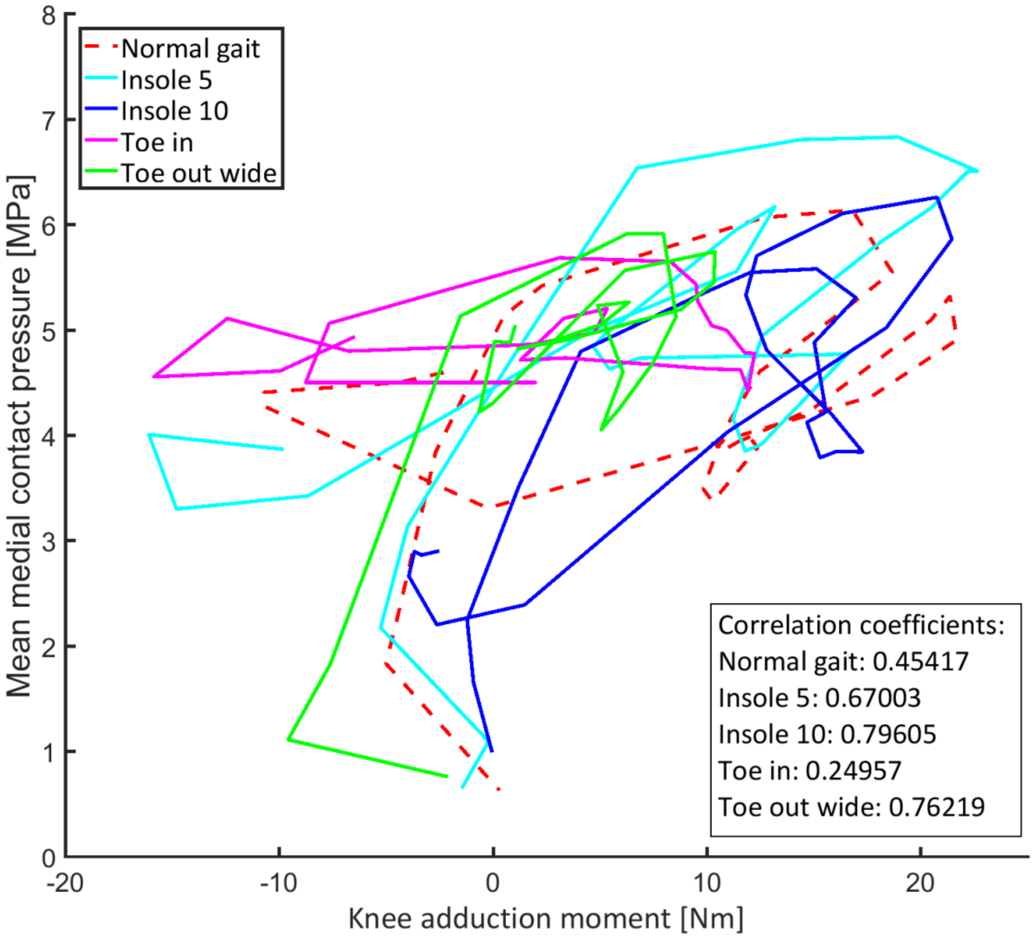

Supplementary Figure C. Mean medial contact pressures in the tibiofemoral contact area vs. knee adduction moment during the stance phase of gait.
